# Supplementary material for: Heat adaptation of phage T7 under an extended genetic code
Source: Virus Evol. 2021 Dec 1;7(2):veab100. doi: 10.1093/ve/veab100 (PMC8923235; doi:10.1093/ve/veab100)
Supplement: veab100_Supp [file veab100_supp.zip › Sequencing_Analysis.zip!/Sequencing_Analysis/S1/evidence/JC_39.html]

BRESEQ :: Evidence


| New junction evidence | | | | | | | | | | | |
| --- | --- | --- | --- | --- | --- | --- | --- | --- | --- | --- | --- |
|  | | seq id | position | reads (cov) | reads (cov) | score | skew | freq | annotation | gene | product |
| \* | ? | NC\_001604.1 | 111 = | 2696 (0.640) | 18 (0.000) | 12/592 | 43.6 | 0.66% | intergenic (–/–) | *–/–* | –/– |
|  | ? | NC\_001604.1 | 121 = | 2794 (0.670) |  |  |  |  | intergenic (–/–) | *–/–* | –/– |
| Rejected: Coverage evenness skew score above cutoff. | | | | | | | | | | | |
| Rejected: Frequency below/above cutoff threshold. | | | | | | | | | | | |

Only 100 of 152 total aligned reads displayed.  

`TGTCTCTCCTTATAGTGAGTTGTATTAATAAAGACAATGATTAAAGGTTATCACTTTATGGCTAACAACAAGGCGTCTTTAGGTCTGGTCTTTATGTAGTGTCTTTAGGTCTGGTCTTTAGGTCTGGTCTTTATGTTTAAACTTTAAGATAGGCGTTGACTTGATGGGTCTTTAGGTGTAGGCTTTAGGTGTTGGCTTTAGGATGGACGTTAGGAGGTGACTTTAGGAGGATACTTTAGGAGACTGTAACAGATAGGGACACAGAGAGACACTCAAGGTAACACCCAAAGACAAACCCAAGGGT‑‑‑‑‑‑‑‑‑‑‑‑‑‑‑‑‑‑‑‑‑‑‑‑‑‑‑‑‑‑‑‑‑‑‑‑‑‑‑‑‑‑‑‑‑‑‑‑‑‑‑‑‑‑‑‑‑‑‑‑‑‑‑‑‑‑‑‑‑‑‑‑‑‑‑‑‑‑‑‑‑‑‑‑‑‑‑‑‑‑‑‑‑‑‑‑‑‑‑‑‑‑‑‑‑‑‑‑‑‑‑‑‑‑‑‑‑‑‑‑‑‑‑‑‑‑‑‑‑‑‑‑‑‑‑‑‑‑‑‑‑‑‑‑‑‑‑‑‑‑‑‑‑‑‑‑‑‑‑‑‑‑‑‑‑‑‑‑‑‑‑‑‑‑‑‑‑‑‑‑‑‑‑‑‑‑‑‑‑‑‑‑‑‑‑‑‑‑‑‑‑‑‑‑‑‑‑‑‑‑‑‑‑‑‑‑‑‑‑‑‑‑‑‑‑‑‑‑‑‑‑‑‑‑‑‑‑‑‑‑‑‑‑‑‑‑‑‑‑‑‑‑‑‑‑‑‑‑‑‑‑‑‑‑‑‑‑‑‑‑‑‑‑‑‑‑‑‑‑‑‑‑‑‑‑‑‑‑‑‑‑‑‑‑‑‑‑‑‑‑‑‑‑‑‑‑‑‑‑‑‑‑  <  NC_001604.1/414‑111`  
`‑‑‑‑‑‑‑‑‑‑‑‑‑‑‑‑‑‑‑‑‑‑‑‑‑‑‑‑‑‑‑‑‑‑‑‑‑‑‑‑‑‑‑‑‑‑‑‑‑‑‑‑‑‑‑‑‑‑‑‑‑‑‑‑‑‑‑‑‑‑‑‑‑‑‑‑‑‑‑‑‑‑‑‑‑‑‑‑‑‑‑‑‑‑‑‑‑‑‑‑‑‑‑‑‑‑‑‑‑‑‑‑‑‑‑‑‑‑‑‑‑‑‑‑‑‑‑‑‑‑‑‑‑‑‑‑‑‑‑‑‑‑‑‑‑‑‑‑‑‑‑‑‑‑‑‑‑‑‑‑‑‑‑‑‑‑‑‑‑‑‑‑‑‑‑‑‑‑‑‑‑‑‑‑‑‑‑‑‑‑‑‑‑‑‑‑‑‑‑‑‑‑‑‑‑‑‑‑‑‑‑‑‑‑‑‑‑‑‑‑‑‑‑‑‑‑‑‑‑‑‑‑‑‑‑‑‑‑‑‑‑‑‑‑‑‑‑‑‑‑‑‑‑‑‑‑‑‑‑‑‑‑‑‑‑‑‑‑‑‑‑‑‑‑‑‑‑‑‑‑‑‑‑‑‑‑‑‑‑‑‑‑‑‑‑‑‑‑‑‑gggtTTGTCTTTGGGTGTTACCTTGAGTGTCTCTCTGTGTCCCTATCTGTTACAGTCTCCTAAAGTATCCTCCTAAAGTCACCTCCTAACGTCCATCCTAAAGCCAACACCTAAAGCCTACACCTAAAGACCCATCAAGTCAACGCCTATCTTAAAGTTTAAACA‑‑‑‑‑‑‑‑‑‑‑‑‑TAAAGACCAGACCTAAAGACCAGACCTAAAGACACTACATAAAGACCAGACCTAAAGACGCCTTGTTGTTAGCCATAAAGTGATAACCTTTAATCATTGTCTTTATTAATACAACTCACTATAAGGAGAGACAACTTA  >  NC_001604.1/121‑419`  

                                                                                                                                                                                                                                                                                                                                                                                                                                                                                                                                                                                                                                          
`TGTCTCTCCTTATAGTGAGTTGTATTAATAAAGACAATGATTAAAGGTTATCACTTTATGGCTAACAACAAGGCGTCTTTCGGTCTGGTCTTTATGTAGTGTCTTTAGGTCTGGTCTTTAGGTCTGGTCTTTATGTTTAAACTTTAAGATAGGCGTTGACTTGATGGGTCTTTAGGTGTAGGCTTTAGGTGTTGGCTTTAGGATGGACGTTAGGAGGTGACTTTAGGAGGATACTTTAGGAGACTGTAACAGATAGGGACACAGAGAGACACTCAAGGTAACACCCAAAGACAATCCCAAG                                                                                                                                                                                                                                                                                                                             >  1:282500/1‑301`  
`TGTCTCTCCTTATAGTGAGTTGTATTAATAAAGACAATGATTAAAGGTTATCACTTTATGGCTAACAACAAGGCGTCTTTAGGTCTGGTCTTTATGTAGTGTCTTTAGGTCTGGTCTTTAGGTCTGGTCTTTATGTTTAAACTTTAAGATAGGCGTTGACTTGATGGGTCTTTAGTTGTAGGCTTTAGGTGTTGGCTTTAGGATGGACGTTAGGAGGTGACTTTAGGAGGATACTTTAGGAGACTGTAACAGATAGGGACACAGAGAGACACCCAAGGTAACACCCAAAGACAAACCCAAG                                                                                                                                                                                                                                                                                                                             >  1:231614/1‑301`  
`TGTCTCTCCTTATAGTGAGTTGTATTAATAAAGACAATGATTAAAGGTTATCACTTTATGGCTAACAACAAGGCGTCTTTAGGTCTGGTCTTTATGTAGTGTCTTTAGGTCTGGTCTTTAGGTCTGGTCTTTATGTTTAAACTTTAAGATAGGCGTTGACTTGATGGGTCTTTAGGTGTAGGCTTTAGGTGTTGGCTTTAGGATGGACGTTAGGAGGTGACTTTAGGAGGATACTTTAGGAGACTGTAACAGATAGGGACACAGAGAGAAACTCAAGGTACCACCCAAAGACAAACCCAAG                                                                                                                                                                                                                                                                                                                             >  1:276864/1‑301`  
`TGTCTCTCCTTATAGTGAGTTGTAGTAATAAAGAAAATGATTAACGGTTAGCACATTATGGCTGAGAAAAAGACGTATTTAGGTCTAGTCTTTATGTAGTGTCTTTAGGTATGGTCTTTAGGTCTGGTCTTTATGTATAAACTTTAAGATAGGGGTTGACTTGATGGGTCTTTAGGTGTAGGCTTTAGTGGTTGGATTTAGTATGGACGTTAGGAGGTGACTTGAGGAGGATACTTTAGGGGACTGTAACAGATAGGGACACAGAGAGACACTCATGGTCACACCCAAAGACAAACCTAAG                                                                                                                                                                                                                                                                                                                             >  1:83421/1‑301`  
 `GTCTCTCCTTATAGTGAGTTGTATTAATAAAGACAATGATTAAAGGTTATCACTTTATGGCTAACAACAAGGCGTCTTTAGGTCTGGTCTTTATGTAGTGTCTTTAGGTCTGGTCTTTAGGTCTGGTCTTTATGTTTAAACTTTAAGATAGGCGTTGACTTGATGGGTCTTTAGGTGTAGGCTTTAGGTGTTGGCTTTAGGATGGACGTTAGGAGGTGACTTTAGGAGGATACTTTAGGAGACTGTAACAGATAGGGACACAGAGAGACACTCAAGGTAACACCCAAAGACAAACCCAAGG                                                                                                                                                                                                                                                                                                                            >  1:569349/1‑301`  
 `GTCTCTCCTTATAGTGAGTTGTATTAATAAAGACAATGATTAAAGGTTATCACTTTATGGCTAACAACAAGGCGTCTTTAGGTCTGGTCTTTATGTAGTGTCTTTAGGTCTGGTCTTTAGGTCTGGTCTTTATGTTTAAACTTTAAGATAGGCGTTGACTTGATGGGTCTTTAGGTGTAGGCTTTAGGTGTTGGCTTTAGGATGGACGTTAGGAGGTGACTTTAGGAGGATACTTTAGGAGACTGTAACAGATAGGGACACAGAGAGACACTCAAGGTAACACCCAAAGACAAACCCAAGG                                                                                                                                                                                                                                                                                                                            >  1:215565/1‑301`  
 `GTCTCTCCTTATAGTGAGTTGTATTAATAAAGACAATGATTAAAGGTTATCACTTTATGGCTAACAACAAGGCGTCTTTAGGTCTGGTCTTTATGTAGTGTCTTTAGGTCTGGTCTTTAGGTCTGGTCTTTATGTTTAAACTTTAAGATAGGCGTTGACTTGATGGGTCTTTAGGTGTAGGCTTTAGGTGTTGGCTTTAGGATGGACGTTAGGAGGTGACTTTAGGAGGATACTTTAGGAGACTGTAACAGATAGGGACACAGAGAGACAATCAAGGAAACACCCAAAGACAAACCCAAGG                                                                                                                                                                                                                                                                                                                            >  1:564621/1‑301`  
 `GTCTCTCCTTATAGTGAGTTGTATTAATAAAGACAATGATTAAAGGTTATCACTTTATGGCTAACAACAAGGCGTCTTTAGGTCTGGTCTTTATGTAGTGTCTTTAGGTCTGGTCTTTAGGTCTGGTCTTTATGTTTAAACTTTAAGATAGGCATTGACTTGATGGGTCTTTAGGTGTAGGCTTTAGGTGTTGGCTTTAGGATGGACGTTAGGAGGTGACTTTAGGAGGATACTTTAGGAGACTGTAACAGTTAGGGACACAGAGAGACAATCAAGGTAACACCCAAAGACAAACCCAAGG                                                                                                                                                                                                                                                                                                                            >  1:43521/1‑301`  
  `tttcaCCTTATAGTGAGTTGTATTATTAATGACAATGATTAAAGGTTATCACTTTATGGCTAACAACAAGGCGTCTTTAGGTCTGGTCTTTAAGTCGTGTCTTTAGGTTTGGTCTTTAGGTCTGGTCTTTATGTTTAAACTTTAAGATAGGCGTTGACTTGATGGGTCTTTAGGTGTAGGCTTTAGGTGTTGGATTTAGGATGGACGTTAGGAGGTGACTTTAGGATGATACTTTAGGAGACTGTAACAGATAGGGACACAGAGAGACACTCAAGGTAACACCCAAAGACAAACCCAAGGG                                                                                                                                                                                                                                                                                                                           <  1:104482/296‑1`  
  `tcaCTCCTTATAGTGAGTTGTGTTAATAAAGACAATGATTAAAGGTTATCACTTTATGGCTAACAACAAGGCGTCTTTAGGTCTAGTCTTTATGTAGTGTCTTTAGGTCTGGTCTTTAGGTCTGGTCTGTATGTTTAAACTTAAAGATAGGCGTTGACTTGATGGGTCTTTAGTTGTAGGCTTTAGGTGTTGGCTTTAGGATGGACGTTAGGAGGTGACTTTAGGAGGATACTTTAGGAGACTGTAACAGATAGGGACACAGAGAGACACTCAAGGTAACACCCAAAGACAAACCCAAGGG                                                                                                                                                                                                                                                                                                                           <  1:604076/298‑1`  
  `gacagCCTTATAGTGAGTTGTATTAATAAAGACAATGATTAAAGGTTATCACTTTATGGCTAACAACAAGGCGTCTTTAGGTCTGGTCTTTATGTAGTGTCTTTAGGTCTGGTCTTTAGGTCTGGTCTTTATGTTTAAACTTTAAGATAGGCGTTGACTTGATGGGTCTTTAGGTGTAGGCTTTAGGTGTTTGCTTTAGGATGGACGTTAGGAGGTGACTTTAGGAGGATACTTTAGGAGACTGTAACAGATAGGGACACAGAGAGACACTCAAGGTAACACCCAAAGACAAACCCAAGGG                                                                                                                                                                                                                                                                                                                           <  1:478779/296‑1`  
  `cagctaCTTATAGTGAGTTGTATTAATAAAGACAATGATTAAAGGTTATCACTTTATGGCTAACAACAAGGCGTCTTTAGGTCTGGTCTTTATGTAGTGTCTTTAGGTCTGGTCTTTAGGTCTGGTCTTTATGTTTAAACTTTAAGATAGGCGTTGACTTGATGGGTCTTTAGGTGTAGGCTTTAGGTGTTGGCTTTAGGATGGACGTTAGGGGGTGACTTTAGGAGGATACTTTAGGAGACTGTAACAGATAGGGACACAGAGAGACACTCAAGGTAACACCCAAAGACAAACCCAAGGG                                                                                                                                                                                                                                                                                                                           <  1:526599/295‑1`  
  `cagCTCCTTATAGTGAGTTGTATTAATAAAGACAATGATTAAAGGTTATCACTTTATGGCTAACAACAAGGCGTCTTTAGGTCTGGTCTTTATGTAGTGTCTTTAGGTCTGATCTTTAGGTCTGGTCTTTATGTTTAAACTTTAGGATAGGCGTTGACTTGATGGGTCTTTAGGTGTAGGCTTTAGGTGTTGGCTTTAGGATGGACGTTAGGAGGTGACTTTAGAAGGATACTTTAGGAGACTGTAACAGATAGGGACACAGAGAGACACTCAAGGTAACACCCAAAGACAAACCCAAGGG                                                                                                                                                                                                                                                                                                                           <  1:4615/298‑1`  
  `caTCTCCTTAATGTTAGGTGTATTAATAAAGACAATAATTAAAGGTTATCACTTTATGGCTAACAACAAGTCGTTTTTAGGTCTGGTCTTTAGGTAGTGTCTTTAGGTCTGGGCTTGAGGTCTGGTCTTTATGTTTAAACTTTAAGATAGGCGTTGACTTGATGGGTCTTTAGGTGTAGGCTTTAGGTGTTGGCTTTAGGATGGACGTTAGGAGGTGACTTTAGGAGGATACTTTAGGAGACTGTAACAGATAGGGACACAGAGAGACACTCAAGGTAACACCCAAAGACAAACCCAAGGG                                                                                                                                                                                                                                                                                                                           <  1:409814/299‑1`  
  `atatgtttataagagacagGTATTAATAAGGACAATGATTAAAGGTTATTACTTTATGGCTAACAACAAGTCGTCTTTAGGTCTAGTTTTTATGTAGTGTCTTTAGGTCTGGTCTTTAGGTCTGGTCTTTATGTTTAAACTTTAAGATAGGCGTTAACTTGATGGGTCTTTAGGTGTAGGCTTTAGGTGTTGGCTTTAAGATGGGCGTTAGGAGGTGACTTTAGGAGGATACTTTAGGAGACTGTAACAGATAGGGACAGAGAGAGACACTCAAGGTAACACCCAAAGACAAACCCAAGGG                                                                                                                                                                                                                                                                                                                           <  1:411627/282‑1`  
  `agatgtgtataagagacagGTATTAATAAAGACAATGATTAAAGGTTATCACTTTATGGCTAACAACAAGGCGTCTTTAGGTCTGGTCTTTATGTAGTGTCTTTAGGTCTGGTCTTTAGGTCTGGTCTTTATGTTTAAACTTTAAGATAGGCGTTGACTTGATGGGTCTTTAGGTGTAGGCTTTAGGTGTTGGCTTTAGGATGGACGTTAGGAGGTGACTTTAGGAGGATACTTTAGGAGACTGTAACAGATAGGGACACAGAGAGACACTCAAGGTAACACCCAAAGACAAACCCAAGGG                                                                                                                                                                                                                                                                                                                           <  1:572319/282‑1`  
  `agatgtgtataagagacagGTATTAATAAAGACAATGATTAAAGGTTATCACTTTATAGCTAACAACAAGGCGTCGTTAGGTCTGGTCTTTATGTAGTGTCTTTAGGTCTGGTCTTTAGGTCTGGTCTTGATGTTTAAACTTTAAGATAGGCGTTGACTTGATGGGTCTTTAGGTGTAGGCTTTAGGTGTTGGCGTTAGGATGGACGTGAGGAGGTGACTTTAGGAGGATACTTTAGGAGACTGTAACAGATAGGGACACAGAGAGACACTCAAGGTAACACCCAAAGACAAACCCAAGGG                                                                                                                                                                                                                                                                                                                           <  1:89828/282‑1`  
  `agatgtgtataagagaaagGTATTAATAAAGACAATGATTAAAGGTTATCACTTTATGGCTAACAACAAGGCGTCTTTAGGTCTGGTCTTTATGTAGTGTCTTTAGTTCTGGTCTTTAGGTCTGGTCTTTATGTTTAAACTTTAAGATAGGCGTTGACTTGATGGGTCTTTAGGTGTAGGCTTTAGGTGTTGGCTTTAGGATGGACG‑TAGGAGGTGACTTTAGGAGGATACTTTAGGAGACTGTAACAGATAGGGACACAGAGAGACACTCAAGGTAACACCCAAAGACAAACCCAAGGGT                                                                                                                                                                                                                                                                                                                          <  1:176767/282‑1`  
  `aagttctTTAAAGTTAGTTTAATTAATAAAACAAATGATTAAAGGTTATCACTTAATGGCTAAAAACAAGGCGTCTTTAGGTCTGGTCTTTATGTAGTTTCTTTAGGTCTGTTCTTTAGGTCTGGTCTTTATGTTTAAACTTTAAGATAGGCGTTGACTTGATGGGTCTTTAGGTGTAGGCTTTAGGTGTTGGCTTTAGGATGGACGTTAGGAGGTGACTTTAGGAGGATACTTTAGGAGACTGTAACAGATAGGGACACAGAGAGACACTCAAGGTAACACCCAAAGACAAACCCAAGGG                                                                                                                                                                                                                                                                                                                           <  1:666020/294‑1`  
  `aagagacagATAGTGAGTTGTATTAATAAAGACAATGATTAAAGGTTATCACTTTATGGATAACAACAAGGCGTCTTTAGGTCTGGTCTTTATGTAGTGTCTTTAGGTCTGGTCTTTAGGTCTGGTCTTTATGTTTAAACTTCAAGATAGGCGTTGACTTGATGGGTCTTTAGGTGTAGGCTTTAGGTGTTGGCTTTAGGATGGACGTTAGGAGGTGACTTTAGGAGGATACTTTAGGAGACTGTAACAGATAGGGACACAGAGAGACACTCAAGGTAACACCCAAAGACAAACCCAAGGG                                                                                                                                                                                                                                                                                                                           <  1:422124/292‑1`  
  `aagagacaTATAGTGAGTTGTATTAATAAAGACAATGATTAAAGGTTATCACTTTATGGCTAACAACAAGGCGTCTTTAGGTCTGGTCTTTATGTAGTGTCTTTAGGTCTGGTCTTTAGGTCTGGTCTTTATGTTTAAACTTTAAGATAGGCGTTGACTTGATGGGTCTTTAGGTGTAGGCTTTAGGTGTTGGCTTTAGGATGGACGTTAGGAGGTGACTTTAGGAGGATACTTTAGGAGACTGTAACAGATAGGGACACAGAGAGACACTCAAGGTAACACCCAAAGACAAACCCAAGGG                                                                                                                                                                                                                                                                                                                           <  1:476885/293‑1`  
  `aacagcttaaaAGTTAGTTGTATTAATAAAGAAAATGATTAAAGGTTATCACCTTATGGTTAACAAGAAGGCGTCGGTAAGTCTGGTCCTGATGTTGTGTCTTGAGGTATGGTCTTTAGGTCTGGTCGTTATGTTTAAACTTTAAGATAGGCGTTGACTTTATGGGTCTTTAGGTGTAGGCTTTAGGTGTTGGCTTTAGGATGGACGTTAGGAGGTGACTGTAGGAGGATACTTTAGGAGACTGTAACAGATAGGGACACAGAGAGACACTCAAGGTAACACCCAAAGACAAACCCAAGGG                                                                                                                                                                                                                                                                                                                           <  1:353118/290‑1`  
  `aacagCCTTGTAGTGAGTTGTATTAATAAAGACAATGATTAAAGGTTATCACTTTATGGCTAACAACAAGGCGTCTTTAGGTCTGGTCATTATGTAGTGTCTTTAGGTCTGGTCTTTAGGTCTGGTCTTTATGTTTAAACTTTAAGATAGGCGTTGACTTGATGGGTCTTTAGGTGTAGGCTTTAGGTGTTGGCTTTAGGATGGAAGTTAGGAGGTGACTTTAGGAGGATACTTTAGGAGACTGTAACAGATATGGACACAGAGAGACACTCAAGGTAACACCCAAAGACAAACCCAAGGG                                                                                                                                                                                                                                                                                                                           <  1:554368/296‑1`  
  `aacagCCTTATAGTGAGTTGTATTAATAAAGACAATGATTAAAGGTTATCACTTTATGGCTAACAACAAGGCGTTTTTAGGTCTGGTCTTTATGTAGTGTCTTTAGGTCTGGTCTTTAGGTCTGGTCTTTATGTTTAAACTTTAAGATAGGCGTTGACTTGATGGGTCTTTAGGTGTAGGCTTTAGGTGTTGGCTTTAGGATGGACGTTAGGAGGTGACTTTAGGAGGATACTTTAGGAGACTGTAACAGATAGGGACACAGAGAGACACTCAAGGTAACACCCAAAGACAAACCCAAGGG                                                                                                                                                                                                                                                                                                                           <  1:472629/296‑1`  
  `TCTCTCTTTATAGTGAGTTGGATTAATAAAGAAAATGATTAAAGGTGATAAATTGATGGATAACAACAAGGCGTCTTTAGGTCTGGTCTTTATGTAGTGTCTGTAGGTCTGGTCTTTAGGTCTGGTTGTTATGTTTAAACTTTAAGATAGGCGTTGACTTGATGGGTCTTTAGGTGTAGGCTTTAGGTGTTGGCTTTAGGATGGACGTTAGGAGGTGACTTTAGGAGGATACTTTAGGAGACTGTAACAGATAGGGACACAGAGAGACACTCAAGGTAACACCCAAAGACAAACCCAAGGG                                                                                                                                                                                                                                                                                                                           <  1:40101/301‑1`  
  `TCTCTCCTTGTAGTGAGTTGTATTAATAAATACAATGATTAAAGGTTATCACTTTATGGCTAACAACAAGTTGTCTTTAGGTCTGGTCTTTATGTAGTGTCTTTAGGTCTGGTCTTTAGGTCTGGTCTTTATGTTTAAACTTTAAGATAGGCGTTGACTTGATGGGTCTTTAGGTGTAGGCTTTAGGTGTTGGCTTTAGGATGGACGTTAGGAGGTGACTTTAGGAGGATACTTTAGGAGACTGTAACAGATAGGGACACAGAGAGACACTCAAGGTAACACACAAAGACAAACCCAAGGG                                                                                                                                                                                                                                                                                                                           <  1:167123/301‑1`  
  `TCTCTCCTTATAGTGAGTTTTATTAATAAAGACATTGATTAAAGGTGATCATTTTATGGCTAACAACAAGGCGTTTTTAGGTCTGGTCTTTATGTAGTGTCTTTAGGTCTGTTCTCTAGGTCTGGTCTTTATGTTTAAACTTTAAGATAGTCGTTGACTTGATGGGTCTTTAGGTGTAGGCTTTAGGTGTTGGCTTTAGGATGGACGTGAGGAGGTGACTTTAGGAGGATACTTTAGGAGACTGTAACAGATCGGGACACAGAGAGACACTCAAGGTAACACACAAAGACAAACCCAAGGG                                                                                                                                                                                                                                                                                                                           <  1:649209/301‑1`  
  `TCTCTCCTTATAGTGAGTTGTATTAATAAAGAGAATGATTAAAGGTTATCACTTTATGGCTAACCACAAGGCGTCTTTAGGTCTGGTCTTTATGTAGTGTCTTTAGTTCTGGTCTTTAGGTCTGGTCTTTATGTTTAAACTTTAAGATAGGCGTTGACTTGATGGGTCTTTAGGTGTAGGCTTTAGGTGTTGGCTTTAGGATGGCCGTTAGGAGGTGACTTTAGGAGGATACTTTAGGAGACTGTAACAGATAGGGACACAGAGAGACACTCAAGGTAACACCCAAAGACAAACCCAAGGG                                                                                                                                                                                                                                                                                                                           <  1:6030/301‑1`  
  `TCTCTCCTTATAGTGAGTTGTATTAATAAAGACAATGATTAAAGGTTATCACTTTATGGCTAACAACAATGCGTCTTTAGGTCTGGTCTTTATGTAGTGTCTTTAGGTCTGGTCTTTAGGTCTGGTCTTTATGTTTAAACTTGAAGATAGGCGTTGACTTGATGGGTCTTTAGGTGTAGGCTTTAGGTGTTGGCTTTAGGATGGACGTTAGGAGGTGACTTTAGGAGGATACTTTAGGAGACTGTAACAGATAGGGACACAGAGAGACACTCAAGGTAACACCCAAAGACAAACCCAAGGG                                                                                                                                                                                                                                                                                                                           <  1:280263/301‑1`  
  `TCTCTCCTTATAGTGAGTTGTATTAATAAAGACAATGATTAAAGGTTATCACTTTATGGCTAACAACAAGGCGTCTTTAGGTCTGGTCTTTATGTAGTGTCTTTAGGTCTGGTCTTTAGGTCTGGTCTTTATGTTTAAACTTTAAGATAGGCGTTGACTTGATGGGTCTTTAGGTGTAGGCTTTAGGTGTTGGCTTTAGGATGGACGTTAGGAGGTGACTTTAGGAGGATACTTTAGGAGACTGTAACTGATAGGGACACAGAGAGACACTCAAGGTAACACCCAAAAACAAACCCAAGGG                                                                                                                                                                                                                                                                                                                           >  1:385441/1‑301`  
  `TCTCTCCTTATAGTGAGTTGTATTAATAAAGACAATGATTAAAGGTTATCACTTTATGGCTAACAACAAGGCGTCTTTAGGTCTGGTCTTTATGTAGTGTCTTTAGGTCTGGTCTTTAGGTCTGGTCTTTATGTTTAAACTTTAAGATAGGCGTTGACTTGATGGGTCTTTAGGTGTAGGCTTTAGGTGTTGGCTTTAGGATGGACGTTAGGAGGTGACTTTAGGAGGATACTTTAGGAGACTGTAACAGATAGGGACACAGAGAGACACTCAAGGTAACACCCAAAGACAAACCCAAGGG                                                                                                                                                                                                                                                                                                                           <  1:90363/301‑1`  
  `TCTCTCCTTATAGTGAGTTGTATTAATAAAGACAATGATTAAAGGTTATCACTTTATGGCTAACAACAAGGCGTCTTTAGGTCTGGTCTTTATGTAGTGTCTTTAGGTCTGGTCTTTAGGTCTGGTCTTTATGTTTAAACTTTAAGATAGGCGTTGACTTGATGGGTCTTTAGGTGTAGGCTTTAGGTGTTGGCTTTAGGATGGACGTTAGGAGGTGACTTTAGGAGGATACTTTAGGAGACTGTAACAGATAGGGACACAGAGAGACACTCAAGGTAACACCCAAAGACAAACCCAAGGG                                                                                                                                                                                                                                                                                                                           <  1:258142/301‑1`  
  `TCTCTCATTATAGTGAGTTGTATTAATAAAGACAATGATTAAAGGTTATCACTTTATGGCTAACAACAAGGCGTCTTTAGGTCTGGTCTTTATGTAGTGTCTTTAGTTCTGGTCTTTAGGTCTGGTCTTTATGTTTAAACTTTAAGATAGGCGTTGACTTGATGGGTCTTTAGGTGTAGGCTTTAGGTGTTGGCTTTAGGATGGACGTTAGGAGGTGACTTTAGGAGGATACTTTAGGAGACTGTAACAGATAGGGACACAGAGAGACACTCAAGGTAACACCCAAAGACAAACCCAAGGG                                                                                                                                                                                                                                                                                                                           <  1:414731/301‑1`  
  `TCTCACCTTATAGTGAGTTGTATTAATAAAGACAATGATTAAAGGTTATCACTTTATGGCTAACAACAAGGCGTCTTTAGGTCTGGTCTTTATGTAGTGTCTTTAGGTCTGGTCTTTAGGTCTGGTCTTTATGTTTAAACTTTAAGATAGGCGTTGACTTGATGGGTC‑TTAGGTGTAGGCTTTAGGTGTTGTCTTTAGGATGGACGTTAGGAGGTGACTTTAGGAGGATACTTTAGGAGACTGTAACAGATAGGGACACAGAGAGACACTCAAGGTAACACCCAAAGACAAACCCAAGGGc                                                                                                                                                                                                                                                                                                                          <  1:590949/301‑2`  
   `tctcactttatgtgtattgttaTAATAAAGGACATGATTTAAGGGTATTAATTTAAGGATAAACAAAAGGCGGTGTTAGGTTTGGTTTTTATGTAGTGTCTTTAGGTCTGGTCTTTAGGTCTGGTCTTTATGTTTAAACTTTAAGATAGGCGTTGACGTGATGGGTCTTTAGGTGTAGGCTTTAGGTGTTGGCTTTAGGATGGACGTTAGGAGGTGACTTTAGGAGGATACTTTAGGAGACTGTAACAGATAGGGACACAGAGAGACACTCAAGGTAACACCCAAAGACAAACCCAAGGGT                                                                                                                                                                                                                                                                                                                          <  1:644486/279‑1`  
   `gatgtgtataagagacagGTATTAATAAAGACAATGATTAAAGGTTATCACTTTATGGCTAACAACAAGGCGTCTTTAGGTCTTGTCTTTATGTAGTGTCTTTAGGTCTGGTCTGTAGGTCTGGTCTTTATGTTTAAACTTTAAGATAGGCGTTGACTTGATGGGTCTTTAGGTGTAGGCTTTAGGTGTTGGCTTTAGGATGGACGTTAGGAGGTGACTTTAGGAGGATACTTTAGGAGACTGTAACAGTTAGGGACACAGAGAGACACTCAAGGTAACACCCAAAGACAAACCAAAGGGT                                                                                                                                                                                                                                                                                                                          <  1:57379/283‑1`  
   `gatgtgtataagagacagGTATTAATAAAGACAATGATTAAAGGTTATCACTTTATGGCTAACAACAAGGCGTCTTTAGGTCTGGTCTTTATGTAGTGTCTTTAGGTCTGGTCTTTAGGTCTGGTCTTTATGTTTAAACTTTAAGATAGGCGTTGACTTGATGGGTCTTTAGGTGTAGGCTTTAGGTGTTGGCTTTAGGATGGACGTTAGGAGGTGACTTTAGGAGGATACTTTAGGAGACTGTAACAGATAGGGACACAGAGAGACACTCAAGGTAACACCCAAAGACAAACCCAAGGGT                                                                                                                                                                                                                                                                                                                          <  1:12121/283‑1`  
   `ctaTCCTTATAGTGAGTTGTATTAATAAAGACAATGAGTAAAGGTTATCACTTTATGGCTAACAACAAGGCGTATGTAGGTCTGGTCTTTATGTAGTGTCTTTAGGTCTGGTCTTTAGGTCTGGTCTTTATGTTTAAACTTTAAGATAGGCGTTGACTTGATGGGTCTTTAGGTGTAGGCTTTAGGTGTTGGCTTTAGGATGGACGTTAGGAGGTGACTTTAGGAGGATACTTTAGGAGACTGTAACAGATAGGGACACAGAGAGACACTCAAGGTAACACCCAAAGACAAACCCAAGGGT                                                                                                                                                                                                                                                                                                                          <  1:262511/298‑1`  
   `cagTCCTTATAGTGAGTTGTATTAATAATGACAATGATTAAAGGTTATCACTTTATGGCTAACAACAAGTCTTCTTGAGTTCTGGTCTTTATGTAGTGTCTTTAGGTCTGGTCTTTAGTTCTGGTCTTTATGTTTAAACTTTAAGATAGGCGTTGACTTTATGGGTCTTTAGGTGTAGGCTTTAGGTGTTGGCTTTAGGATGGACGTTAGGAGGTGACTTTAGGAGGATACTTTAGGAGACTGTAACAGATAGGGACACAGAGAGACACTCAAGGTAACACCCAAAGACAAACCCAAGGGT                                                                                                                                                                                                                                                                                                                          <  1:376169/298‑1`  
   `acagCCTTATAGTGATTTGTATGAATAAATACAATGATTAAAGTTGATCACTTTATGGCTAACAACAAGGCGTCTTTAGGTCTGGGCTTTATGTAGTGTCTTTAGGTCGGGTCTTTAGGTCTGGTCTTTATGTTTAAACTTTAAGATAGGCGTTTACTTGATGGGTCTTTAGGTGTAGGCTTTAGGTGTTGGCTTTAGGATGGACGTTAGGAGGTGACTTTAGGAGGATACTTTAGGAGACTGTAACAGATAGGGACACAGAGAGACACTCAAGGTAACACCCAAAGACAAACCCAAGGGT                                                                                                                                                                                                                                                                                                                          <  1:320926/297‑1`  
   `acagCCTTATAGGGAGTTGTATTAATAAAGACAATGATTAAAGGTTATCACTTTATGGCTAACAACAAGGCGTCTTTAGGTCTGGTCTTTATGTAGTGTCTTTAGGTCTGGTCTTTAGGTCTGGTCTTTATGTTTAAACTTTAAGATAGGCGTTGACTTGATGGGTCTTTAGGTGTAGGCTTTAGGTGTTGGCTTTAGGATGGACGTTAAGAGGTGACTTTAGGAGGATACTTTAGGAGACTGTAACAGATAGGGACACAGAGAGACACTCAAGGTAACACCCAAAGACAAACCCAAGGGT                                                                                                                                                                                                                                                                                                                          <  1:219838/297‑1`  
   `aatgtgtataagagacagGTATTAATAAAGACAATGATTAAAGGTTATCACTTTATGGCTAACAACAAGGCGTCTTTAGGTCTTGTCTTTATGTAGTGTCTTTAGGTCTGGTCTTTAGGTCTGGTCTTTATGTTTAAACTTTAAGATAGGCGTTGACTTGATGGGTCTTTAGGTGTAGGCTTTAGGTGTTGGCTTTAGGATGGACGTTAGGAGGTGACTTTAGGAGGATACTTTAGGAGACTGTAACAGATAGGGACACAGAGAGACACTCAAGGTAACACCCAAAGACAAACCCAAGGGT                                                                                                                                                                                                                                                                                                                          <  1:292729/283‑1`  
   `CTCTCCTTATAGTGAGTTGTATTAATAAAGACAATGATTAAAGGTTATCACTTTATGGCTAACAACAAGGCGTCTTTAGGTCTGGTCTTTATGTAGTGTCTTTAGGTCTGGTCTTTAGGTCTGGTCTTTATGTTTAAACTTTAAGATAGGCGTTGACTTGATGGGTCTTTAGGTGTAGGCTTTAGGTGTTGGCTTTAGGATGGACGTTAGGAGGTGACTTTAGGAGGATACTTTAGGAGACTGTAACAGATAGGGACACAGAGAGACACTCAAGGTAACACCCAAAGACAAACCCAAGGGT                                                                                                                                                                                                                                                                                                                          <  1:94734/301‑1`  
   `CTCTCCTTATAGTGAGTTGTATTAATAAAGACAATGATTAAAGGTTATCACTTTATGGCTAACAACAAGGCGTCTTTAGGTCTGGTATTTATGTAGTGTCTTTAGGTCTGGTCTTTAGGTCTGGTCTTTATGTTTAAACTTTAAGATAGGCGTTGACTTGATGGGTCTTTAGGTGTAGGCTTTAGGTGTTGGCTTTAGGATGGACGTTAGGAGGTGACTTTAGGAGGATACTTTAGGAGACTGTAACAGATAGGGACACAGAGAGACACTCAAGGTAACACCCAAAGACAAACCCAAGGGa                                                                                                                                                                                                                                                                                                                          >  1:221090/1‑300`  
   `CTCTCCTTAAAGTGAGTTGTATTAAAAAAGACAATGATTAAAGGTTATCACTTTATGGCTAACAACAAGGCGTTTTTAGGTCTGGTCTTTATGTAGTGTCTTTAGGTCTGGTCTTTAGGTCTGGTCTTTATGTTTAAACTTTAAGATAGGCGTTGACTTGATGGGTCTTTAGGGGTAGGCTTTAGGTGTTGGCTTTAGGATGGACGTTAGGAGGTGACTTTAGGAGGATACTTTAGGAGACTGTAACAGATAGGGACACAGAGAGACACTCAAGGTAACACCCAAAGACAAACCCAAGGGT                                                                                                                                                                                                                                                                                                                          <  1:79733/301‑1`  
     `CTCCTTATAGTTAGTTGTATTAATAAAGACAATGATTAAAGGTTCTCACTTTATGGCTAACAACAAGGCGTCTTTAGGTCTGGTCTTTATGTAGTGTCTTTAGTTCTGGTCTTTTGGTCTGGTCTTTTTGTTTAAACTTTAAGATAGGCGTTGACTTGATGGGTCTTTAGGTGTCGGCTTTAGGTGTTGGTTTTAGGCGGGACGTTAGTAGGTGACTTTAGGAGGATACTTTCGGGGACTGTAATCGATAGGGGCACAGAGAGACATCCAAGGTCACACCCAAAGACAACACCACGGGTaa                                                                                                                                                                                                                                                                                                                        >  1:538508/1‑299`  
     `CTCCTTATAGTGAGTTGTATTAATAAAGACAATGATTAAAGGTTATCACTTTATGGCTAACAACAAGGCGTCTTTAGGTCTGGTCTTTATGTAGTGTCTTTAGGTCTGGTCTTTAGGTCTGGTCTTTATGTTTAAACTTTAAGATAGGCGTTGACTTGATGGGTCTTTAGGTGTAGGCTTTAGGTGTTGGCTTTAGGATGGACGTTAGGAGGTGACTTTAGGAGGATACTTTAGGAGACTGTAACAGATAGGGACACAGAGAGACAGTCAAGGTAACACCCAAAGAAAAACCCAAGGGgca                                                                                                                                                                                                                                                                                                                        >  1:638453/1‑298`  
     `CTCCTTATAGTGAGTTGTATTAATAAAGACAATGATTAAAGGTTATCACTTTATGGCTAACAACAAGGCGTCTTTAGGTCTGGTCTTTATGTAGTGTCTTTAGGTCTGGTCTTTAGGTCTGGTCTTTATGTTTAAACTTTAAGATAGGCGTTGACTTGATGGGTCTTTAGGTGTAGGCTTTAGGTGTTGGCTTTAGGATGGACGTTAGGAGGTGACTTTAGGAGGATACTTTAGGAGACTGTAACAGATAGGGACACAGAGAGAAACTCAAGGTAACACCCAAAGACAAACCCAAGGGatg                                                                                                                                                                                                                                                                                                                        >  1:46855/1‑298`  
      `TCCTTATAGTGAGTTGTATTAATAAAGACAATGATTAAAGGTTATCACTTTATGGCTAACAACAAGGCGTCTTTAGGTCTGGTCTTTATGTAGTGTCTTTAGGTCTGGTCTTTAGGTCTGTTCTTTATGTTTAAACTTTAAGATAGGCGTTGACTTGATGGGTCTTTAGGTGTAGGCTTTAGGTGTTGGCTTTAGGATGGACGTTAGGAGGTGACTTTAGGAGGATACTTTAGGAGACTGTAACAGATAGGGACACAGAGAGACAATCAAGGTAACACCCAAAGCAAAACCCAAGGGTact                                                                                                                                                                                                                                                                                                                       >  1:425503/1‑298`  
      `TCCTTATAGTGAGTTGTATTAATAAAGACAATGATTAAAGGTTATCACTTTATGGCTAACAACAAGGCGTCTTTAGGTCTGGTCTTTATGTAGTGTCTTTAGGTCTGGTCTTTAGGTCTGGTCTTTATGTTTAAACTTTAAGATAGGCGTTGACTTGATGGTTCTTTAGGTGTAGGCTTTAGGTGTTGGCTTTAGGATGGATGTTAGGAGGTGACTTCAGGAGGATACTTTAGGAGACTGTAACAGATAGGGACACAGAGAGACACTCAAGGTAACACCCAAAGACAAACCCAAGGGTaat                                                                                                                                                                                                                                                                                                                       >  1:304616/1‑298`  
      `TCCTTATAGTGAGTTGTATTAATAAAGACAATGATTAAAGGTTATCACTTTATGGCTAACAACAAGGCGTCTTTAGGTCTGGTCTTTATGTAGTGTCTTTAGGTCTGGTCTTTAGGTCTGGTCTTTATGTTTAAACTTTAAGATAGGCGTTGACTTGATGGGTCTTTAGGTGTAGGCTTTAGGTGTTGGTTTTAGGATGGACGTTAGGAGGTGACTTTAGGAGGATACTTTAGGAGACTGTAACAGATAGGGACACAGAGAGACACTCAAGGTAACACCCCAAGACAAACCCAAGGGacat                                                                                                                                                                                                                                                                                                                       >  1:468913/1‑297`  
      `TCCTTATAGTGAGTTGTATTAATAAAGACAATGATTAAAGGTTATCACTTTATGGCTAACAACAAGGCGTCTTTAGGTCTGGTCTTTATGTAGTGTCTTTAGGTCTGGTCTTTAGGTCTGGTCTTTATGTTTAAACTTTAAGATAGGCGTTGACTTGATGGGTCTTTAGGTGTAGGCTTTAGGTGTTGGCTTTAGGATGGACGTTAGGAGGTGACTTTAGGAGGATACTTTAGGAGACTGTAACAGATAGGGACACAGAGAGACACTCAAGGTAACACCCAAAGACAAACCCAAGGttcaa                                                                                                                                                                                                                                                                                                                       >  1:330430/1‑296`  
      `TCCTTATAGTGAGTTGTATTAATAAAGACAATGATTAAAGGTTATCACTTTATGGCTAACAACAAGGCGTCTTTAGGTCTGGTCTTTATGTAGTGTCTTTAGGTCTGGTCTTTAGGTCTGGTCTTTATGTTTAAACTTTAAGATAGGCGTTGACTTGATGGGTCTTTAGGTGTAGGCTTTAGGTGTTGGCTTTAGGATGGACGTTAGGAGGTGACTTTAGGAGGATACTTTAGGAGACTGTAACAGATAGGGACACAGAGAGACACTCAAGGTAACACCCAAAGACAAACCCAAGGGctgt                                                                                                                                                                                                                                                                                                                       >  1:451211/1‑297`  
      `TCCTTATAGTGAGTTGTATTAATAAAGACAATGATTAAAGGTTATCACTTTATGGCTAACAACAAGGCGTCTTTAGGTCTGGTCTTTATGTAGTGTCTTTAGGTCTGGTCTTTAGGTCTGGTCTTTATGTTTAAACTTTAAGATAGGCGTTGACTTGATGGGTCTTTAGGTGTAGGCTTTAGGTGTTGGCTTTAGGATGGACGTTAGGAGGTGACTTTAGGAGGATACTTTAGGAGACTGTAACAGATAGGGACACAGAGAGACACTCAAGGTAACACCCAAAGACAAACCAAAGGGccat                                                                                                                                                                                                                                                                                                                       >  1:267589/1‑297`  
      `TCCTTATAGTGAGTTGTATTAATAAAGACAATGATTAAAGGTTATCACTTTATGGCTAACAACAAGGCGTCTTTAGGTCTGGTCTTTATGTAGTGTCTTTAGGTCTGGTCTTTAGGTCTGGTCTTTATGTTTAAACTTTAAGATAGGCGTTGACTTGATGGGTCTTTAGGTGTAGGCTTTAGGTGTTGGCTTTAGGATGGACGGTAGGAGGTGACTTTAGGAGGATTCTTTAGGAGACTGTAACAGGTGGTGACGCAGAGAGACACTCAAAGTACCGCCCAAAGACAAACCCAAGGGctgt                                                                                                                                                                                                                                                                                                                       >  1:472980/1‑297`  
      `TCCTTATAGTGAGTTGTATTAATAAAGACAATGATTAAAGGTTATCAATTTATGGCTAACAACAAGGCGTCTTTAGGTCTGGTCTTTATGTAGTGTCTTTAGGTCTGGTCTTTAGGTCTGGTCTTTATGTTTAAACTTTAAGATAGGCGTTGACTTGATGGGTCTTTAGGTGTAGGTTTTAGGTGTTGGCTTTAGGATGGACGTTAGGAGGTGACTTTAGGGGGATACTTTAGGAGACTGTAACAGATAGGGACACAGAGAGAAACTAAAGTTAAAACCCAAAGAAAAACACAAGGGTaaa                                                                                                                                                                                                                                                                                                                       >  1:426822/1‑298`  
       `CCTTATAGTGAGTTGTATTAATAAAGACAATGATTAAAGGTTATCACTTTATGGCTAACAACAAGGCGTCTTTAGGTCTGGTCTTTATGTAGTGTCTTTAGGTCTGGTCTTTAGGTCTGGTCTTTATGTTTAAACTTTAAGATAGGCGTTGACTTGATGGGTCTTTAGGTGTAGGCTTTAGGTGTTGGCTTTCGGATGGACGTTAGGAGGTGACTTTAGTAGGATACTTTAGGAGACTGTAACAGATAGGGACACAGAGAGACACTCAAGGTCACACCCAAAGACAAACCCAAGGcctgtc                                                                                                                                                                                                                                                                                                                      >  1:458313/1‑295`  
       `CCTTATAGTGAGTTGTATTAATAAAGACAATGATTAAAGGTTATCACTTTATGGCTAACAACAAGGCGTCTTTAGGTCTGGTCTTTATGTAGTGTCTTTAGGTCTGGTCTTTAGGTCTGGTCTTTATGTTTAAACTTTAAGATAGGCGTTGACTTGATGGGTCTTTAGGTGTAGGCTTTAGGTGTTGGCTTTAGGATGGACGTTAGGAGGTGACTTTAGGAGGATACTTTAGGAGACTGTAACAGATAGGGACACAGAGAGACACTCAAGGTAACACCCAAAGACAAACCCAAGGGTaatc                                                                                                                                                                                                                                                                                                                      >  1:579572/1‑297`  
        `CTTATAGTGAGTTGTATTAATAAAGACAATGATTAAAGGTTATCACTTTATGGCTAACAACAAGGCGTCTTTAGGTCTGGTCTTTATGTAGTGTCTTTAGGTCTGGTCTTTAGGTCTGGTCTTTATGTTTAAACTTTAAGATAGGCGTTGACTTGATGGGTCTTTAGGTGTAGGCTTTAGGTGTTGGCTTTAGGATGGACGTTAGTAGGTGACTTTAGGAGGAGACTTTAGGAGACTGTAACAGATAGGGACACAGAGAGCAACTAAAGGTAACAACCAAGAACAAAACCAAGctgtctct                                                                                                                                                                                                                                                                                                                     >  1:62106/1‑293`  
        `CTTATAGTGAGTTGTATTAATAAAGACAATGATTAAAGGTTATCACTTTATGGCTAACAACAAGGCGTCTTTAGGTCTGGTCTTTATGTAGTGTCTTTAGGTCTGGTCTTTAGGTCTGGTCTTTATGTTTAAACTTTAAGATAGGCGTTGACTTGATGGGTCTTTAGGTGTAGGCTTTAGGTGTTGGCTTTAGGATGGACGTTAGGAGGTGACTTTAGGAGGATACTTTAGGAGACTGTAACAGATAGGGACACAGAGAGACACTCAAGGTAACACCCAAAGACAAACCCAAGGGTCTGTC                                                                                                                                                                                                                                                                                                                     >  1:153973/1‑301`  
           `ATAGTGAGTTGTATTAATAAAGACAATGATTAAAGGTTATCACTTTATGGCTAACAACAAGGCGTCTTTAGGTCTGGTCTTTATGTAGTGTCTTTAGGTCTGGTCTTTAGGTCTGGTCTTTATGTTTAAACTTTAAGATAGTCGTTGACTTGATGGGTCTTTAGGTGTAGGCTTTAGGTGTTGGCTTTAGGATGGACGTTAGGAGGTGACTTTAGGAGGATACTTTAGGAGACTGTAACAGATAGGGACACAGAGAGACACTCAAGGTAACACCCAAAGACAAACCCAAGGGctgtctctt                                                                                                                                                                                                                                                                                                                  >  1:217031/1‑292`  
           `ATAGTGAGTTGTATTAATAAAGACAATGATTAAAGGATATCACTTTATGGCTAACAACAAGGCGTCTTTAGGTCTGGTATTTATGTAGTGTCTTTAGGTCTGGTCTTTAGGTCTGGTCTTTATGTTTAAACTTTAAGATAGGCGTTAACTTGATGGGTCTTTAGGTGTAGGCTTTAGGTGTTGGCTTTAGGATGGACGTTAGGAGGTGACTTTAGAAGGATAGTTTAGGAGACTGTAACAGATAGGGACACAGAAAGACACTCAAGGTAAAACCAAAAGAAAACACCAAGagacgctgtca                                                                                                                                                                                                                                                                                                                  >  1:315892/1‑290`  
                `GAGTTGTATTAATAAAGACAATGATTAAAGTTTATCACTTTATGGCTAACAACAAGGCGTCTTTAGGTCTGGTCTTTATGTAGTGTCTTTAGGTCTGGTCTTTAGGTCTGGTCTTTATGTTTAAACTTTAAGATAGGCGTTGACTTGATGGGTCTTTAGTTGTAGGCTTTAGGTGTTGGCTTTAGGATGGACGTTAGGAGGTGACTTTAGGAGGATACTTTAGGAGACTGTAACAGATAGGGACACAGAGAGACACTCAAGGTAACACCCAAAGACAAACCCAAGGGTCTGTCTcttatac                                                                                                                                                                                                                                                                                                             >  1:57624/1‑294`  
                     `GTATTAATCAAGACAATGATTAAAGGTTATCACTTTATGGCTAACAACAAGGCGTCTTTAGGTCTGGTCTTTATGTAGTGTCTTTAGGTCTGGTCTTTAGGTCTGGTCTTTATGTTTAAACTTTAAGATAGGCGTTGACTTGATGGGTCTTTAGGTGTAGGCTTTAGGTGTTGGCTTTAGGATGGACGTTAGGAGGTGACTTTAGGAGGATACTTTAGGAGACTGTAACAGATAGGGACACAGAGAGACACTCAAGGTAACACCCAGAGACAAACCCAAGGGctgtctcttatacacatct                                                                                                                                                                                                                                                                                                        >  1:609104/1‑282`  
                     `GTATTAATAAAGACAATGATTAAAGGTTATCACTTTATGGCTAACAACAAGGCGTCTTTAGGTCTGGTCTTTATGTAGTGTCTTTAGGTCTGGTCTTTAGGTCTGGTCTTTATGTTTAAACTTTAAGATAGGCGTTGACTTGATGGGTCTTTAGGTGTAGGCTTTAGGTGTTGGCTTTAGGATGGACGTTCGGAGGTGACTTTAGGAGGATACTTTAGGAGACTGTAACAGTTAGGGACACAGAGAGACACACAAGGTAACACCCAAAGACAAACCCAAGGGTCTGTCTTTtataaacatt                                                                                                                                                                                                                                                                                                        >  1:356396/1‑291`  
                     `GTATTAATAAAGACAATGATTAAAGGTTATCACTTTATGGCTAACAACAAGGCGTCTTTAGGTCTGGTCTTTATGTAGTGTCTTTAGGTCTGGTCTTTAGGTCTGGTCTTTATGTTTAAACTTTAAGATAGGCGTTGACTTGATGGGTCTTTAGGTGTAGGCTTTAGGTGTTGGCTTTAGGATGGACGTTAGGAGGTGACTTTAGGAGGATACTTTAGGAGACTGTAACAGATAGGGACACAGAGAGACATTCAAGGTAACACCCAAAGACAAACCAAAGGGTCTGTCTcttatacacatt                                                                                                                                                                                                                                                                                                        >  1:36525/1‑289`  
                     `GTATTAATAAAGACAATGATTAAAGGTTATCACTTTATGGCTAACAACAAGGCGTCTTTAGGTCTGGTCTTTATGTAGTGTCTTTAGGTCTGGTCTTTAGGTCTGGTCTTTATGTTTAAACTTTAAGATAGGCGTTGACTTGATGGGTCTTTAGGTGTAGGCTTTAGGTGTTGGCTTTAGGATGGACGTTAGGAGGTGACTTTAGGAGGATACTTTAGGAGACTGTAACAGATAGGGACACAGAGAGACACTCAAGGTAACACCCAAAGACAAACCCAAGGGTCTGTCccttatacacatc                                                                                                                                                                                                                                                                                                        >  1:574670/1‑288`  
                          `AATAAAGACAATGATTAAAGGTTATCACTTTATGGCTAACAACAAGGCGTCTTTAGGTATGGTCTTTATGTCGTGTCTTTAGGTCTGGTCTTTAGGTCTGGTCTTTATGTTTAAACTTTAAGATATGCGTTGACTTGATGGGTCTTGTGGTGTAGGCTTTAGGTGTTGGATCTAGGATGGACGTTAGTAGGTTACTTTCGGAGGATACTTTAGGAGTCTGTAAAAGTTAGGGACACAGAGAGACAGTCAAGGTAACACTCAAAGGAAAACACAAGGagaatacccaacgcgaaggcaaccc                                                                                                                                                                                                                                                                                                   >  1:349816/1‑276`  
                          `AATAAAGACAATGATT‑AAGGTTATCACTTTATGGCTAACAACAAGGCGTCTTTAGGCCTGGTCTTTATGTAGTGTCTTTAGGTCTGGTCTTTAGGTCTGGTCTTTATGTTTAAACTTTAAGATAGGCGTTGACTCGCTGGGTCTTTAGGTGTAGGCTTTAGGCGTTGGCTTTAGGATGGACGTTCGGAGGTGACTTTAGGAGGATACTTTAGGAGACTGAAACAGTTAGGGTCACAGAGAGACACTCAAGGTAATACCTAAAGACAAAACCAACGGTaattcaccaccattcaggctgttt                                                                                                                                                                                                                                                                                                  >  1:463556/1‑277`  
                                      `GATTAAAGGTTATCACTTTATGGCTAACAACAAGGCGTCTTTAGGTCTGGTCTTTATGTAGTGTCTTTAGGTCTGGTCTTTAGGTCTGGTCTTTATGTTTAAACTTTAAGATAGGCGTTGACTTGATGGGTCTTTAGGTGTAGGCTTTAGGTGTTGGCTTTAGGATGGACGTTAGGAGGTGACTTTAGGAGGATACTTTAGGAGACTGTAACAGATAGGGACACAGAGAGACACTAAAGGTAACACCCAAAGACAAACCTAAGGGACTGTCTcttatacacatctccgagcccacgagaca                                                                                                                                                                                                                                                                                       >  1:557658/1‑272`  
                                                                             `tttgtataatagacagatgtactggttgTAGGTCTGGTCTTTAGGTCAGGTCTTTATGTTTAAATTTTAAGATAGGCGTTGACTTTATGGGTCTTTAGGTGTAGGCTTTAGGTGTTGGCTTTAGAATGGACGTTAGGAGTTGACTTGAGGAGGATACTTTAGGAGACTGTAACAGATAGGGACACAGAGAGACACTCAAGGTAACACCCAAAGACAAACCCAAGGGTTTGTCTTTGGGTGTTACCTTGAGTGTCTCTCTGTGTCCCTATCTGTTACAGTCTCCTAAAGTATCCTCCTAAAG                                                                                                                                                                                                                                                <  1:559135/273‑1`  
                                                                                         `tttgATGTAGTGTCTTTAGGTCTGGTCTTTTGGTCGTGTCTCTAGTTTTGCACTTTAAGATCGGCGTTGACTTGATTGGTCTTTAGGTGTAGGCTTGAGTTGTTTGCTTTAGGATGGACGTTAGGAAGTGACTTTATGAGTATACTTTAGGAGACTGTAACAGATAGGGACACAGAGAGACACTCAAGGTAACACCCAAAGACAAACCCAATGGTTTGTCTTTGGGTGTTACCTTGAGTGTCTCTCTGTGTCCCTATCTGTTACAGTCTCCTAAAGTATCCTCCTAAAGTCACCTCCTAAC                                                                                                                                                                                                                                    <  1:443862/297‑1`  
                                                                                                                                                                                                                                                   `CTGTAACAGATAGGGACACAGAGAGACACTCAAGGTAACACCCA‑‑‑‑‑‑‑‑‑‑‑AAGGGTTTGTCTTTGGGTGTTACCTTGAGTGTCTCTCTGTGTCCCTATCTGTTACAGTCTCCTAAAGTATCCTCCTAAAGTCACCTCCTAACGTCCATCCTAAAGCCAACACCTAAAGCCTACACCTAAAGACCCATCAAGTCAACGCCTATCTTAAAGTTTAAACA‑‑‑‑‑‑‑‑‑‑‑‑‑TAAAGACCAGACCTAAAGACCAGACCTAAAGACACTACATAAAGACCAGACCTAAATACGCCTTTTTGTTAGCCATAAAGTGATAACCTT                                                  >  1:416700/1‑301`  
                                                                                                                                                                                                                                                                                    `ctcgtagatgtgtataagagacagGGGTTTTGCTTTGGGTGTTACCTTGAGTGTCTCTCTGTGTCCCTATCTGTTACAGACTCCTAAAGTATCCTCCTAAAGTCACCTCCTAACGTCCATCCTAAAGCAAACACCTAAAGCCTACTCCTAAAGACCCATCAAGTCAACGCCTATATTAAAGTTTAAACA‑‑‑‑‑‑‑‑‑‑‑‑‑TAAAGACCAGACCTAAAGACCAGACCTAAAGACACTACATAAAGACCAGACCTAAAGACGCCTTGTTGTTAGCCATAAAGTGATAACCTTTAATCATTGTCTTTATTAATAC                            <  1:335366/277‑1`  
                                                                                                                                                                                                                                                                                    `ctcggagatgtgtataagagacagGGGTTTGTCTTTGGGTGTTACCTTGAGTGTCTCTCTGTGTCCCTATCTGTTACAGTCTCCTAAAGTATCCTCCTAAAGTCACCTCCTAACGTCCATCCTAAAGCCAACACCTAAAGCCTACACCTAAAGACCCATCAAGTCAACGCCTATCTTAAAGTTTAAACA‑‑‑‑‑‑‑‑‑‑‑‑‑TAAAGACCAGACCTAAAGACCAGACCTAAAGACACTACATAAAGACCAGACCTAAAGACGCCTTGTTGTTAGCCATAAAGTGATAACCTTTAATCATTGTCTTTATTAATAC                            <  1:372834/277‑1`  
                                                                                                                                                                                                                                                                                              `gtgtataagggacAGGGATTATCTTTGGGTGTTACCTGAAATGTCTCTCTGTGTCCCTAGCTGTTACAGTCTCCTAAAGTATCCACCTAAAGTCACCTCCTAACATCCATCCTAAAGCCAACACCTAAAGCCTACACCTAAAGACCCATAAAGTCAACGCCTATCTTAAAGTTTAAACA‑‑‑‑‑‑‑‑‑‑‑‑‑AAAAGACCAGACCTAAAGACCAGACCTAAAGACACTACATAAAGACCAGACCTAAAGACGCCTTGTTGTTAGCCATAAAGTGATAACCTTTAATCATTGTCTTTATTAATACAACTCACTAT                  <  1:617013/288‑1`  
                                                                                                                                                                                                                                                                                                  `taagagacagGGGTTTGTCTTTGGGTGTTACCTTGAGTGTCTCTCTGTGTCCCTATCTGTTACAGTCTCCTAAAGTATCCTCCTAAAGTCACCTCCTAACGTCCATCCTAAAGCCAACACCTAAAGCCTACACCTAAAGACCCATCAAGTCAACGCCTATCTTAAAGTTTAAACA‑‑‑‑‑‑‑‑‑‑‑‑‑TAAAGACCAGACCTAAAGACCAGACCTAAAGACACTACATAAAGACCAGACCTAAAGACGCCTTGTTGTTAGCCATAAAGTGATAACCTTTAATCATTGTCTTTATTAATACAACTCACTATAAGG              <  1:142616/291‑1`  
                                                                                                                                                                                                                                                                                                  `gatcacacttaGGTTTGTCTTTGGGTGTTACCTTGAGTGTCTCTCTGTGTCCCTATCTGTTACAGTCTCCTAAAGTATCCTCCTAAAGTCACCTCCTAACGTCCATCCTAAAGCCAACACCTAAAGCCTACACCTAAAGACCCATCAAGTCAACGCCTATCTTAAAGTTTAAACA‑‑‑‑‑‑‑‑‑‑‑‑‑TAAAGACCAGACCTAAAGACCAGACCTAAAGACACTACATAAAGACCAGACCTAAAGACGCCTTGTTGTTAGCCATAAAGTGATAACCTTTAATCATTGTCTTTATTAATACAACTCACTATAAGG              <  1:510478/290‑1`  
                                                                                                                                                                                                                                                                                                   `taagagagAGGGTGTGTCTGTGGGTGTTACCGTGATTGTCTCACTGTGTCAATATCTGTTACAGTCTCCTAAAGTATCCTCCTAAAGTCACCACATAACGTCCAGCCTAAATCCAACACCTAAAGACTACACCTAAAGACCCATCAAGTCAACGCCTAACTTAAAGTTTAAACA‑‑‑‑‑‑‑‑‑‑‑‑‑TAAAGACCAGACCTAAAGACCAGACCTAGAGACACTACATAAAGACCAGACCTAAAGACGCCTTGTTGTTAGCCATAAAGTGATAACCTTTAATCATTGTCTTTATTAATACAACTCACTATAAGGA             <  1:605029/293‑1`  
                                                                                                                                                                                                                                                                                                   `atgactctgaGGTTTGTCTTTGGGTTGTACCTTGAGTGTCTCTCTGTGTCCCTAACTGTTACAGTCTCCTAAAGTAACCTCCTAAAGTCACCTCCTAACGGCCATCCTAAAGCCAACCCCTAAATCCTACACCTAAAGACCCATCAAGTCAACGCCTATCTTAAAGTTTAAACA‑‑‑‑‑‑‑‑‑‑‑‑‑TAAAGACCAGACCTAAAGACCAGACCTAAAGACACTACATAAAGACCAGACCTAAAGACGCCTTGTTGTTAGCCATAAAGTGATAACCTTTAATCATTGTCTTTATTAATACAACTCACTATAAGGA             <  1:450651/291‑1`  
                                                                                                                                                                                                                                                                                                   `ataagagacatGTTTGTCTTTAGTTTTTACTTTGAGTGTCTCTCTGTGTCCCTTTCTTTTCAAGTCTCCTAAAGTATCCTCCTAAAGTCACCTCCTACCGTCCATCCTAAAGCCAACACCTAAAGCCTACACCTAAAGACCCATCAAGTCAACGCCTATCTTAAAGTTTAAACA‑‑‑‑‑‑‑‑‑‑‑‑‑TAAAGACCAGTCCTAAAGACCAGACCTAAAGACACTACATAAAGACCAGACCTAAAGACGCCTTGTTGTTAGCCATAAAGTGATAACCTTTAATCATTGTCTTTATTAATACAACTCACTATAAGGA             <  1:329099/290‑1`  
                                                                                                                                                                                                                                                                                                   `aaagcactcgcGTTTGTCCTTGGGTGTTAACTTAAGTGTCTCTCTGTGTCCCCATCTGTAACAGTCTCCTAAAGAATCCTCCTAAAGTCAACTCCTAACGTTCATCCTAAAGCCAACACCTAAAGACTACACCTAAAGACACATCAAGTCAACGACAATATTAAAGTTAAAAGA‑‑‑‑‑‑‑‑‑‑‑‑‑TAAAGACAAGAACAAAAGACCAGACATAAAGACACTACATAAAGACCAGAACTAAAGACGCCTTGTTTTTAGCCATAAAGTGATAACCTTTAATCATTGTCTTTATTAATACAACTCACTATAAGGA             <  1:143340/290‑1`  
                                                                                                                                                                                                                                                                                                    `tgaacgttgtGTTTGTCTTTGGGTGTTACATTGAGTCTCTTTCTGTGTCCCTATCTGTTACAGTCTCCTAAAGTATCCTCCTAAAGTCACCTCCTAACTTCCATCCTAAAGCCAACACCTAAAGCCTACACCTAAAGACCCATCAAGTCAACGCCTATCTTAAAGTTTAAACA‑‑‑‑‑‑‑‑‑‑‑‑‑TAAAGACCAGACCTAAAGACCCGACCTAAAGACACTACATAAAGACCAGACCTAAAGCCGCCTTGTTGTTAGCCATAAAGTGATAACCTTTAATCATTGTCTTTATTAATACAACTCACTATAAGGAG            <  1:155014/291‑1`  
                                                                                                                                                                                                                                                                                                    `agagacagGGGTTTGTCTTTGGGTGTTACCTTGAGTGTCTCTCTGTGTCCCTATCTGTTACAGTCTCCTAAAGTATCCTCCTAAAGTCACCTCCTAACGTCAATCCTAAAGCCAACACCTAAAACCTACACCTAAAGACCCATCAAGTCAACGCCTATCTTAAAGTTTAAACA‑‑‑‑‑‑‑‑‑‑‑‑‑TAAAGACCAGACCTAAAGACCAGACCAAAAGACACTACATAAAGACCAGACCTAAAGACGCCTTGTTGTTAGCCATAAAGTGATAACCTTTAATCATTGTCTTTATTAATACAACTCACTATAAGGAG            <  1:526907/293‑1`  
                                                                                                                                                                                                                                                                                                    `agagacagGGGTTTGTCTTTGGGTGTTACCTTGAGTGTCTCTCAGTGTCCCTATCTGTTACAGTCTCCTAAAGTATCCTCCTAAAGTCACCTCCTAACGTCCATCCTAAAGCCAACACCTAAAGCCTACACCTAAAGACCCATCAAGTCAACGCCTATCTTAAAGTTTAAACA‑‑‑‑‑‑‑‑‑‑‑‑‑TAAAGACCAGACCTAAAGACCAGACCTAAAGACACTACATAAAGACCAGACCTAAAGACGCCTTGTTGTTAGCCATAAAGTGATAACCTTTAATCATTGTCTTTATTAATACAACTCACTATAAGGAG            <  1:552011/293‑1`  
                                                                                                                                                                                                                                                                                                    `agagacagGGGTTTGACTTTGGGTGTTACCTTGAGTGTCTCTCTGTGTCCTTATCTGTTACAGTCTCCTAAAGTATCCTCCTAAAGTCACCTCCTAACGTCCATCCTAAAGCCAACACCTAAAGCCTACACCTAAAGACCCATCAAGTCAACGCCTATCTTAAAGTTTAAACA‑‑‑‑‑‑‑‑‑‑‑‑‑TAAAGACCAGACCTAAAGACCAGACCTAAAGACACTACATAAAGACCAGACCTAAAGACGCCTTGTTGTTAGCCATAAAGTGATAACCTTTAATCATTGTCTTTATTAATACAACTCACTATAAGGAG            <  1:35322/293‑1`  
                                                                                                                                                                                                                                                                                                           `gaGGTATGTCTTGGGGTGTTACCTGGAGTGACTCTCTATGTCCGTATCTGTTACAGTCACCTAAAATATCCACCTAAAGACACCGCCTAACGTCCAACCTAAGGACAACGCCTAAAGCCTAAGACTAAAGATCCATAAAGTCAACACCTATCATAAAGTTTAAACA‑‑‑‑‑‑‑‑‑‑‑‑‑TAAAGACCAGACCTAAAGACCAGACCTAAAGACACTACAGAAAGACCAGACCTAAAGACGCCTTGTTGTTAGCCATAAAGTGATAACCTTTAATCATTGTCTTTATTAATACAACTCACTATAAGGAGAGACAAC     <  1:334010/299‑1`  
                                                                                                                                                                                                                                                                                                            `GGGTTTGTCTTTGGGTGTTACCTTGAGTGTCTCTCTGTGTCCCTATCTGTTACAGTCTCCTAAAGTATCCTCCTAAAGTCACCTCCTAACGTCCATCCTAAAGCCAACACCTAAAGCCTACACCTAATGACCCATCAAGTCAACGCCTATCTTAAAGTTTAAACA‑‑‑‑‑‑‑‑‑‑‑‑‑TAAAGACCAGACCTAAAGACCAGACCTAAAGACACTACATAAAGACCAGACCTAAAGACGCCTTGTTGTTAGCCATAAAGTGATAACCTTTAATCATTGTCTTTATTAATAACAATTACTATAAactgcctcttat    >  1:137461/1‑289`  
                                                                                                                                                                                                                                                                                                            `GGGTTTGTCTTTGGGTGTTACCTTGAGTGTCTCTCTGTGTCCCTATCTGTTACAGTCTCCTAAAGTATCCTCCTAAAGTCACCTCCTAACGTCCATCCTAAAGCCAACACCTAAAGCCTACACCTAAAGACCCATCAAGTCAACGCCTATCTTAAAGTTTACACA‑‑‑‑‑‑‑‑‑‑‑‑‑TAAAGACCAGACCTAAAGACCAGACCTAAAGACACTACATAAAGACCAGACCTAAAGACGCCTTGTTGTTAGCCATAAAGTGATAACCATTAATCATTGTCTTTATTAATACAACTAACTATAAGctgactcttat    >  1:137452/1‑290`  
                                                                                                                                                                                                                                                                                                            `GGGTTTGTCTTTGGGTGTTACCTTGAGTGTCTCTCTGTGTCCCTATCTGTTACAGTCTCCTAAAGTATCCTCCTAAAGTCACCTCCTAACGTCCATCCTAAAGCCAACACCTAAAGCCTACACCTAAAGACCCATCAAGTCAACGCCTATCTTAAAGTTTAAACA‑‑‑‑‑‑‑‑‑‑‑‑‑TAACGACCAGACCTAAAGACCAGACCTAAAGACACTACATAAAGACCAGACCTAAAGACGCCTTGTTGTTAGCAATAAAGTGATAACCTTTAATCATTGTCTTTATTAATACAACTCACTATAAGGAGAGACAACT    >  1:505630/1‑301`  
                                                                                                                                                                                                                                                                                                            `GGGTTTGTCTTTGGGTGTTACCTTGAGTGTCTCTCTGTGTCCCTATCTGTTACAGTCTCCTAAAGTATCCTCCTAAAGTCACCTCCTAACGTCCATCCTAAAGCCAACACCTAAAGCCTACACCTAAAGACCCATCAAGTCAACGCCTATCTTAAAGTTTAAACA‑‑‑‑‑‑‑‑‑‑‑‑‑TAAAGACCAGACCTAAAGACCAGACCTAAAGACACTACATAAAGACCAGACCTAAAGACGCCTTGTTGTTAGCCATAAAGTGATAACCTTTAATCATTGTCTTTATTAATACAACTCctgtctcttatacacatct    >  1:531996/1‑282`  
                                                                                                                                                                                                                                                                                                            `GGGTTTGTCTTTGGGTGTTACCTTGAGTGTCTCTCTGTGTCCCTATCTGTTACAGTCTCCTAAAGTATCCTCCTAAAGTCACCTCCTAACGTCCATCCTAAAGCCAACACCTAAAGCCTACACCTAAAGACCCATCAAGTCAACGCCTATCTTAAAGTTTAAACA‑‑‑‑‑‑‑‑‑‑‑‑‑TAAAGACCAGACCTAAAGACCAGACCTAAAGACACTACATAAAGACCAGACCTAAAGACGCCTTGTTGTTAGCCATAAAGTGATAACCTTTAATCATTGTCTTTATTAATACAACTCACTATAAGGAGAGACActg    >  1:23226/1‑298`  
                                                                                                                                                                                                                                                                                                            `GGGTTTGTCTTTGGGTGTTACCTTGAGTGTCTCTCTGTGTCCCTATCTGTTACAGTCTCCTAAAGTATCCTCCTAAAGTCACCTCCTAACGTCCATCCTAAAGCCAACACCTAAAGCCTACACCTAAAGACCCATCAAGTCAACGCCTATCTTAAAGTTTAAACA‑‑‑‑‑‑‑‑‑‑‑‑‑TAAAGACCAGACCTAAAGACCAGACCTAAAGACACTACATAAAGACCAGACCTAAAGACGCCTTGTTGTTAGCCATAAAGTGATAACCTTTAATCATTGTCTTTATTAATACAACTCACTATAAGGAGAGACAACT    >  1:458485/1‑301`  
                                                                                                                                                                                                                                                                                                            `GGGTTTGTCTTTGGGTGTTACCTTGAGTGTCTCTCTGTGTCCCTATCTGTTACAGTCTCCTAAAGTATCCTCCTAAAGTCACCTCCTAACGTCCATCCTAAAGCCAACACCTAAAGCCTACACCTAAAGACCCATCAAGTCAACGCCTATCTTAAAGTTTAAACA‑‑‑‑‑‑‑‑‑‑‑‑‑TAAAGACCAGACCTAAAGACCAGACCTAAAGACACTACATAAAGACCAGACCTAAAGACGCCTTGTTGTTAGCCATAAAGTGATAACCTTTAATCATTGTCTTTATTAATACAACTCACTATAAGAAACGACAACT    >  1:103162/1‑301`  
                                                                                                                                                                                                                                                                                                            `GGGTTTGTCTTTGGGTGTTACCTTGAGTGTCTCTCTGTGTCCCTATCTGTTACAGTCTCCTAAAGTATCCTCCTAAAGTCACCTCCTAACGTCCATCCTAAAGCCAACACCTAAAGCCTACACCTAAAGACCCATCAAGTCAACGCCTATCTTAAAGTTTAAACA‑‑‑‑‑‑‑‑‑‑‑‑‑TAAAGACCAGACCTAAAGACCAGACCTAAAGACACTACATAAAGACCAGACCTAAAAACGCCTTGTTGTTAGCCATAAAGTGATAACCTTTAATCATTGTCTTTATTAATACAACTCACTATAAGGAGAGACAACT    >  1:257060/1‑301`  
                                                                                                                                                                                                                                                                                                            `GGGTTTGTCTTTGGGTGTTACCTTGAGTGTCTCTCTGTGTCCCTATCTGTTACAGTCTCCTAAAGTATCCTCCTAAAGTCACCTCCTAACGTCAATCCTAAAGCCAACACCTAAAGCCTACACCTAAAGACCCATCAAGTCAACGCCTATCTTAAAGATTAAACA‑‑‑‑‑‑‑‑‑‑‑‑‑TAAAGACCAGACCTAAAGACCAGACCTAAAGACACTACATAAAGACCAGACCTAAAGACGCCTTGTTGTTATCCATAAAGTGATAACCTTTAATCATTGTCTTAATTAATACAACTCACTATAAGGAGAGACAAat    >  1:398376/1‑299`  
                                                                                                                                                                                                                                                                                                            `GGGTTTGTCTTTGGGTGTTACCATGAGTGTCTCTCTGTGTCCCTATCTGTTACAGTCTCCTAAAGTATCCTCCTAAAGTCACCTCCTAACGTCCATCCTAAAGCCAACACCTAAAGCCTACACCTAAAGACCCATCAAGTCAACGCCTATCTTAAAGTTTAAACA‑‑‑‑‑‑‑‑‑‑‑‑‑TAAAGACCAGACCTAAAGACCAGACCTAAAGACACTACATAAAGACCAGACCTAAAAACGCATTGTGGTTAGCCATAAAGTGATAACCTTTAATAATTGTCTTTATTAATACAACTCACTATAAGGAGAGACAACT    >  1:385819/1‑301`  
                                                                                                                                                                                                                                                                                                             `GGTTTGTCTTTGGGTGTTACCTTGATTGTCTCTCTGTGTCTCTATCTGTTACAGTCTCCTAAAGTATCCTCCTAAAGTCACCTCCTAACGTCCATCCTAAAGCCAACACCTAAAGCCTACACCTAAAACCCCATCAAGTCAACGCCTATCTTCCAGTTTTAACA‑‑‑‑‑‑‑‑‑‑‑‑‑TAATGACCACACCTAAAGAACAGACCTACAGACACTAAATAAAGACCCGCCCTAAAGACGCCTTGTTGTTAGCTCTAAAGTGATAACCTTTATTCCTTGTCTTTATTAATACCACTCctaacatgggcagacaattt   >  1:125388/1‑281`  
                                                                                                                                                                                                                                                                                                              `GTTTGTCTTTGGGTGTTACCTTGAGTGTCTCTCTGTGTCCCTATCTGTTACAGTCTCCTAAAGTATCCTCCTAAAGTCACCTCCTAACGTCCATCCTAAAGCCAACACCTAAAGCCTACACCTAAAGACCCATCAAGTCAACGCCTATCTTAAAGTTTAAACA‑‑‑‑‑‑‑‑‑‑‑‑‑TAAAGACCAGACCTAAAGACAAGACCTAAAGACACTACATAAAGACCAGACCTAAAGACGCCTTGTTGTTAGCCATAAAGTGATAACCTTTAATCATTGTCTTTATTAATACAACTCACTATAAGGAGAGACAACTTA  >  1:349569/1‑301`  
                                                                                                                                                                                                                                                                                                               `TTTGTCTTTGGGTGTTACCTTGAGTGTCTCTCTGTGTCCCTATCTGTTACAGTCTCCTAAAGTATCCTCCTAAAGTCACCTCCTAACGTCCATCCTAAAGCCAACACCTAAAGCCTACACCTAAAGACCCATCAAGTCAACGCCTATCTTAAAGTTTAAACATAAAGACCAGACCTAAAGACCAGACCTAAAGACCAGACCTAAAGACACTACATAAAGACCAGACCTAAAGACGCCTTGTTGTTAGCCATAAAGTGATAACCTTTAATCATTGTCTTTATTAATACAACTCACTATAAGG              <  1:547942/301‑1`  

                                                                                                                                                                                                                                                                                                                                                                                                                                                                                                                                                                                                                                          
`TGTCTCTCCTTATAGTGAGTTGTATTAATAAAGACAATGATTAAAGGTTATCACTTTATGGCTAACAACAAGGCGTCTTTAGGTCTGGTCTTTATGTAGTGTCTTTAGGTCTGGTCTTTAGGTCTGGTCTTTATGTTTAAACTTTAAGATAGGCGTTGACTTGATGGGTCTTTAGGTGTAGGCTTTAGGTGTTGGCTTTAGGATGGACGTTAGGAGGTGACTTTAGGAGGATACTTTAGGAGACTGTAACAGATAGGGACACAGAGAGACACTCAAGGTAACACCCAAAGACAAACCCAAGGGT‑‑‑‑‑‑‑‑‑‑‑‑‑‑‑‑‑‑‑‑‑‑‑‑‑‑‑‑‑‑‑‑‑‑‑‑‑‑‑‑‑‑‑‑‑‑‑‑‑‑‑‑‑‑‑‑‑‑‑‑‑‑‑‑‑‑‑‑‑‑‑‑‑‑‑‑‑‑‑‑‑‑‑‑‑‑‑‑‑‑‑‑‑‑‑‑‑‑‑‑‑‑‑‑‑‑‑‑‑‑‑‑‑‑‑‑‑‑‑‑‑‑‑‑‑‑‑‑‑‑‑‑‑‑‑‑‑‑‑‑‑‑‑‑‑‑‑‑‑‑‑‑‑‑‑‑‑‑‑‑‑‑‑‑‑‑‑‑‑‑‑‑‑‑‑‑‑‑‑‑‑‑‑‑‑‑‑‑‑‑‑‑‑‑‑‑‑‑‑‑‑‑‑‑‑‑‑‑‑‑‑‑‑‑‑‑‑‑‑‑‑‑‑‑‑‑‑‑‑‑‑‑‑‑‑‑‑‑‑‑‑‑‑‑‑‑‑‑‑‑‑‑‑‑‑‑‑‑‑‑‑‑‑‑‑‑‑‑‑‑‑‑‑‑‑‑‑‑‑‑‑‑‑‑‑‑‑‑‑‑‑‑‑‑‑‑‑‑‑‑‑‑‑‑‑‑‑‑‑‑‑‑  <  NC_001604.1/414‑111`  
`‑‑‑‑‑‑‑‑‑‑‑‑‑‑‑‑‑‑‑‑‑‑‑‑‑‑‑‑‑‑‑‑‑‑‑‑‑‑‑‑‑‑‑‑‑‑‑‑‑‑‑‑‑‑‑‑‑‑‑‑‑‑‑‑‑‑‑‑‑‑‑‑‑‑‑‑‑‑‑‑‑‑‑‑‑‑‑‑‑‑‑‑‑‑‑‑‑‑‑‑‑‑‑‑‑‑‑‑‑‑‑‑‑‑‑‑‑‑‑‑‑‑‑‑‑‑‑‑‑‑‑‑‑‑‑‑‑‑‑‑‑‑‑‑‑‑‑‑‑‑‑‑‑‑‑‑‑‑‑‑‑‑‑‑‑‑‑‑‑‑‑‑‑‑‑‑‑‑‑‑‑‑‑‑‑‑‑‑‑‑‑‑‑‑‑‑‑‑‑‑‑‑‑‑‑‑‑‑‑‑‑‑‑‑‑‑‑‑‑‑‑‑‑‑‑‑‑‑‑‑‑‑‑‑‑‑‑‑‑‑‑‑‑‑‑‑‑‑‑‑‑‑‑‑‑‑‑‑‑‑‑‑‑‑‑‑‑‑‑‑‑‑‑‑‑‑‑‑‑‑‑‑‑‑‑‑‑‑‑‑‑‑‑‑‑‑‑‑‑‑gggtTTGTCTTTGGGTGTTACCTTGAGTGTCTCTCTGTGTCCCTATCTGTTACAGTCTCCTAAAGTATCCTCCTAAAGTCACCTCCTAACGTCCATCCTAAAGCCAACACCTAAAGCCTACACCTAAAGACCCATCAAGTCAACGCCTATCTTAAAGTTTAAACA‑‑‑‑‑‑‑‑‑‑‑‑‑TAAAGACCAGACCTAAAGACCAGACCTAAAGACACTACATAAAGACCAGACCTAAAGACGCCTTGTTGTTAGCCATAAAGTGATAACCTTTAATCATTGTCTTTATTAATACAACTCACTATAAGGAGAGACAACTTA  >  NC_001604.1/121‑419`

  

Alignment Legend

Aligned base mismatch/match (shaded by quality score): `ATCG/ATCG` `< 3 ≤` `ATCG/ATCG` `< 10 ≤` `ATCG/ATCG` `< 15 ≤` `ATCG/ATCG` `< 31 ≤` `ATCG/ATCG` `< 38 ≤` `ATCG/ATCG`

Unaligned base: `atcg`    Masked matching base: `atcg`    Alignment gap: `‑`    Deleted base: `‑`

Reads not counted as support for junction

read\_name Not counted due to insufficient overlap past the breakpoint.

read\_name Not counted due to not crossing MOB target site duplication.
